# Health Tract, No. 126

**Published:** 1863-03

**Authors:** 


HEALTH TRACT, No. 126.

### CUTE THINGS.

1. PUT the exact "fare" in the lining of your hat, if you are about to travel in car or omnibus on a miserably cold day, when every change of position is disagreeable, thus obviating the necessity of taking off your gloves, unbuttoning your coat, searching your pockets, making change, and getting chilled; if a lady, carry the money under the edge of your glove.

2. If you are enough of a gentleman to feel obliged to give up your seat in a car to any thing in the shape of a petticoat, whether to mistress or maid, whether to a grandmother or to sweet seventeen, whether to a dowager or a market-woman, take your seat as near the forward part of the vehicle as possible, then your gallantry will be the last to be tried, and the least likely to be challenged.

3. If you want a pair of boots or shoes made to order, and wish to be certain of as easy a fit as that of an old shoe, put on two pair of thick, woolen socks before your measure is taken.

4. If, like a wise sailor, you wish to have "all taut" when the terrible and inevitable financial storm comes sweeping over the nation, within a year after the war closes, sell on the spot whatever is necessary to pay off every dollar of your present indebtedness, and invest all your surplus, be it great or small, in solid land, in fee, without the incumbrance of a single copper cent; the next day begin to retrench in all articles of necessary family expenditure, and let every luxury be banished from your memory as completely as if it had never existed.

5. If you want to avoid being drawn into the common vortex of financial ruin by friends and relations, as dishonest in reality as they are reckless, never indorse for a dime without your wife's written consent, and have placed in letters, golden and large, over the mantle of the family room, and require it to be daily read aloud by each member of the family in turn, just before you go to business after breakfast, the fifteenth verse of the eleventh chapter of Proverbs.

6. If you want to know certainly whether the young lady you think of addressing is a fairy or a fury, tread on her skirt in the street, when she is not aware of your being within a mile of her, and "take an observation" of that face, usually "divine," at the instant of its being turned full upon you. If, out of any thousand ladies promenading the street, you wish to make a selection for a wife who shall combine taste, tidiness, and a true economy, walk behind and notice if in shawl or dress, mantilla, cloak, or what not, there are creases, grease-spots, specks of dried mud, or lint, or string, or feather; if you do, let her go, for creases show that she huddles her garments away, because too lazy to fold them up carefully; a grease-spot proves that she will flop herself down any where, consulting personal ease in preference to all other considerations; and any woman who recklessly runs the risk of soiling a garment irretrievably, rather than take the pains to turn her head half round to see whether she is not about sitting on a lump of butter or in a pool of tobacco-juice, is utterly unworthy of a husband, and is as destitute of any true moral principle as she is of innate purity. A dried speck of mud or piece of lint shows she is a hypocrite or a slouch, as it proves that she is careful only of such parts of her apparel as she thinks most likely to be seen.

7. If you wish the great happiness and the inestimable blessing of being always in good health down to a serene old age, learn while young to take care of that "good constitution" with which a benign Creator has intrusted you.

8. If you have a tremendous moustache, and want to eat bread and molasses, put the bread in first and the molasses afterward.

9. If you want to "prove" the best friend you have, ask him to lend you some money.

10. If you want a burglar to wake you up, put your wash-basin under the door-lock, and draw the key half out; then the slightest touch from the outside imitates a racket among the crockery, opportune to an extreme.

